# Optimized bacterial expression of a synthetic BRIL antibody

**DOI:** 10.1107/S2053230X26001548

**Published:** 2026-03-17

**Authors:** Benjamin F. Cooper, Georgia L. Isom

**Affiliations:** a Sir William Dunn School of Pathology University of Oxford OxfordOX1 3RE United Kingdom; Institut Pasteur de Montevideo, Uruguay

**Keywords:** cryo-EM, X-ray crystallography, BRIL, synthetic antibodies, fiducials

## Abstract

The use of monoclonal fragments antigen binding (Fabs) is a prevalent methodology facilitating protein structure determination via both crystallography and cryo-EM. The development of a synthetic Fab against the BRIL domain improved the accessibility of this approach, providing a general fiducial applicable to any protein of interest via the simple curation of a BRIL fusion protein. Here, we document the generation of a T7 Express Δ*cybC* strain allowing contaminant-free bacterial expression of the synthetic anti-BRIL Fab BAG2. We also report the crystal structure of BAG2 in complex with native cytochrome *b*_562_, a complex arising from expression in canonical *Escherichia coli* strains.

## Abstract 2

Here, we document the generation of a T7 Express Δ*cybC* strain allowing contaminant-free expression of the anti-BRIL Fab BAG2. We also report the crystal structure of BAG2 in complex with native cytochrome *b*_562_, a complex arising from expression in canonical *Escherichia coli* strains.

## 1. Introduction

Single-particle analysis cryo-electron microscopy (SPA cryoEM) has become the primary workhorse for high-resolution protein structure elucidation in recent years owing to its low sample requirements and its wider accommodation of heterogeneous samples and/or complexes (De Zorzi *et al.*, 2016 ▸; Chua *et al.*, 2022 ▸). Nevertheless, macromolecule size remains a fundamental limitation within SPA cryoEM, with substantially fewer high-resolution reconstructions performed for macromolecules below 100 kDa; thus, certain protein classes remain underrepresented.

SPA cryoEM presents an attractive proposition for the structural study of integral membrane proteins, which typically exhibit more incompatibilities with X-ray crystallography or NMR. Nonetheless, integral membrane proteins are typically smaller than 100 kDa; thus, several techniques to append membrane proteins, increasing their applicability for cryoEM, have been developed.

Monoclonal fragments antigen binding (Fabs) present one such methodology to assist the structural elucidation of small membrane proteins. Initially Fabs were utilized to assist membrane-protein crystallization, whereby their large polar surface area facilitates crystal contact formation (Kermani, 2021 ▸). Subsequently Fabs were deployed as fiducials in cryoEM, artificially increasing the particle mass by ∼50 kDa and facilitating image alignment, enabling the high-resolution elucidation of several membrane proteins below the 100 kDa threshold (Wu *et al.*, 2012 ▸; Walsh *et al.*, 2018 ▸; Kim *et al.*, 2019 ▸; Shionoya *et al.*, 2024 ▸; Pan *et al.*, 2020 ▸).

The generation of Fabs against each protein of interest presents a substantial bottleneck in their application to nascent projects. Nevertheless, a recently developed panel of synthetic Fabs raised against the BRIL domain offer universal fiducials which may be applied to any target via assembly of a protein–BRIL fusion (Mukherjee *et al.*, 2020 ▸; Tsutsumi *et al.*, 2020 ▸; Sverak *et al.*, 2024 ▸; Luo *et al.*, 2025 ▸; Zhang *et al.*, 2025 ▸).

We sought to apply this methodology to a small membrane protein of interest within our laboratory; however, we observed the anti-BRIL Fab (BAG2) to co-purify with the soluble cytochrome *b*_562_ when grown in typical *Escherichia coli* expression strains. Here, we report the generation of a T7 Express Δ*cybC* strain, offering an alternative bacterial system for the expression of BAG2 which completely prevents the co-purification of cytochrome *b*_562_.

## 2. Materials and methods

### 2.1. Overexpression and purification of BAG2

*E. coli* expression strains [BL21-Gold, Rosetta (DE3), C43 (DE3) Pro^+^, T7 Express and T7 Express *ΔcybC*] were transformed with a pRH2.2 expression vector harbouring the synthetic, anti-BRIL Fab BAG2 (Mukherjee *et al.*, 2020 ▸). Cultures were grown at 37°C in 2×YT medium supplemented with 100 µg ml^−1^ carbenicillin and 1 m*M* MgSO_4_ until the OD_600_ reached 0.6. Protein expression was induced by the addition of 1 m*M* isopropyl β-d-1-thiogalactopyranoside (IPTG) and continued for 4 h at 37°C.

Cells were harvested via centrifugation at 6000*g* for 15 min. The cell pellets were resuspended in an appropriate volume of 50 m*M* Tris, 500 m*M* NaCl pH 8 supplemented with cOmplete EDTA-free protease-inhibitor cocktail tablets (Roche). The cells were lysed via four passes through an Emulsiflex-C3 cell disruptor (Avestin). The cell lysate was incubated at 58°C for 30 min to denature bacterial proteins before cell debris was removed via centrifugation at 50 000*g* for 1 h at 4°C. The resulting supernatant was filtered through a 0.22 µm syringe filter and bound to a HiTrap Protein L column (Cytiva) pre-equilibrated in 50 m*M* Tris, 500 m*M* NaCl pH 8. The column was washed with 10 column volumes (CV) of 50 m*M* Tris, 500 m*M* NaCl pH 8 and the protein was eluted with 0.1 *M* acetic acid. Fractions containing BAG2 were pooled and loaded onto a 1 ml RESOURCE S column (Cytiva) pre-equilibrated in 50 m*M* sodium acetate pH 5.0. The column was washed with 5 CV of 50 m*M* sodium acetate pH 5.0 and the protein was eluted using a linear gradient to 100% 50 m*M* sodium acetate, 2 *M* NaCl pH 5.0 over 25 CV. Fractions containing BAG2 were dialysed overnight at 4°C against 20 m*M* Tris, 150 m*M* NaCl pH 8, concentrated in a 10 kDa molecular-weight cutoff (MWCO) concentrator (Sartorius) and snap-frozen for later use.

### 2.2. BAG2–BRIL fusion-protein complex formation

BAG2 was combined with BRIL fusion protein at a 1.2× molar excess, incubated on ice for 60 min and injected onto a Superose 6 10/300 Increase column (Cytiva). Successful complex formation was established via the analysis of elution fractions by SDS–PAGE.

### 2.3. Crystallization and structure determination of the cytochrome *b*_562_–BAG2 complex

Fractions corresponding to the cytochrome *b*_562_–BAG2 complex were pooled and concentrated to 5–10 mg ml^−1^ in a 50 kDa MWCO concentrator (Sartorius). Crystallization screens were prepared by mixing 100 nl cytochrome *b*_562_–BAG2 complex and 100 nl precipitant solution. The complex crystallized in space group *P*6_5_ (*a* = 88.72, *b* = 88.72, *c* = 162.51 Å, α = 90, β = 90, γ = 120°) in a precipitant solution from the Morpheus screen (Gorrec, 2009 ▸) comprising 0.1 *M* MOPS/HEPES–Na pH 7.5, 0.03 *M* bromide, 0.03 *M* fluoride, 0.03 *M* imidazole, 12.5%(*w*/*v*) PEG 1000, 12.5%(*w*/*v*) PEG 3350, 12.5%(*v*/*v*) MPD. Diffraction data were collected on beamline I04 at Diamond Light Source (DLS). Data reduction and processing was performed using the *xia*2–*DIALS* pipeline (Winter *et al.*, 2018 ▸; Winter, 2010 ▸).

The crystal structure of the native cytochrome *b*_562_–BAG2 complex was solved by molecular replacement with a modified version of the BAG2–BRIL domain structure (PDB entry 6cbv) in which the BRIL domain was replaced with cytochrome *b*_562_, using *Phaser* (McCoy *et al.*, 2007 ▸). The resulting model was refined using *Phenix* (Liebschner *et al.*, 2019 ▸) interspersed with manual inspection and adjustment in *Coot* (Emsley & Cowtan, 2004 ▸). Crystallization, diffraction data-collection and processing and refinement statistics are given in Tables 1 ▸, 2 ▸ and 3 ▸, respectively.

### 2.4. Knockout generation

The T7 Express *ΔcybC* strain was derived from T7 Express (NEB) using lambda Red recombination, as described previously (Datsenko & Wanner, 2000 ▸). Briefly, the *aph* cassette was amplified from donor plasmid pKD4 using primers oBFC307 and oBFC308 (Table 4 ▸) and the linear product introduced to the parental T7 Express strain carrying the Red helper plasmid pKD46. Transformants were initially selected by growth on LB agar supplemented with 50 µg ml^−1^ kanamycin and the *ΔcybC::aph* allele verified by colony PCR using flanking primers oBFC309 and oBFC310 (Table 4 ▸). Finally, the *ΔcybC::aph* allele was P1 transduced into a fresh T7 Express background as described previously (Thomason *et al.*, 2007 ▸).

## 3. Results

### 3.1. BAG2 co-purifies with cytochrome *b_562_*

**Figure 1:**
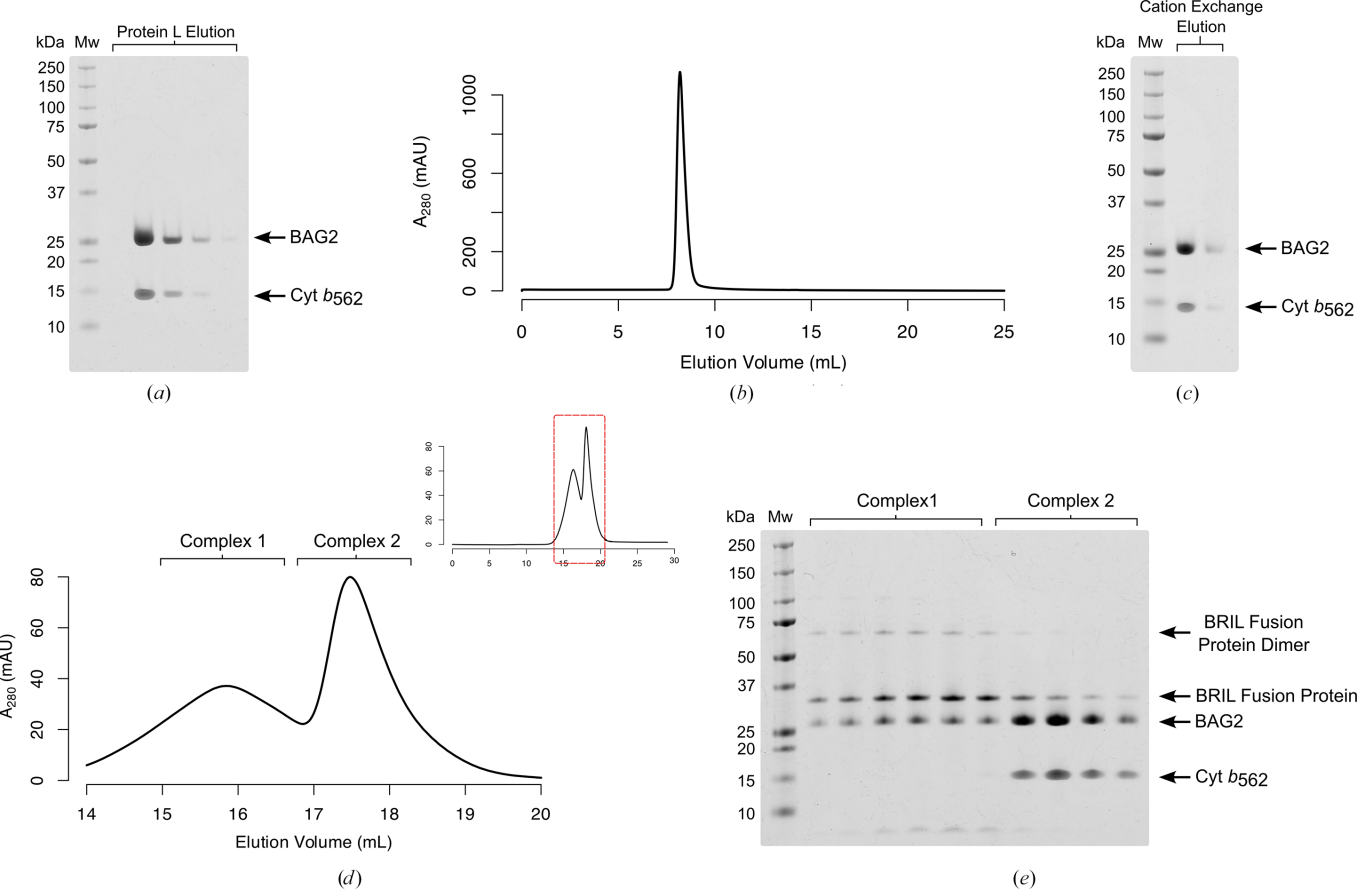
BAG2 co-purifies with a 15 kDa protein which competes for BRIL binding. (*a*) SDS–PAGE of Protein L elution fractions following BAG2 overexpression in BL21-Gold indicating co-purification of a 15 kDa protein, later confirmed as cytochrome *b*_562_. (*b*) Cation-exchange trace of pooled Protein L elution fractions following BAG2 overexpression in BL21-Gold. (*c*) SDS–PAGE of cation-exchange fractions following BAG2 overexpression in BL21-Gold, indicating that BAG2 maintains a stable complex with the 15 kDa protein later identified as cytochrome *b*_562_ throughout purification. (*d*) Region of interest from a size-exclusion chromatography trace following incubation of a BRIL fusion protein (∼37 kDa) with BAG2 purified from BL21-Gold indicating two distinct complexes with different retention volumes. The full trace, highlighting the region of interest, is provided as an inset at the top right. (*e*) SDS–PAGE of size-exclusion chromatography fractions following incubation of a BRIL fusion protein (∼37 kDa) with BAG2 purified from BL21-Gold. Fractions corresponding to complex 1 comprise the expected BRIL fusion protein–BAG2 complex, whilst complex 2 fractions comprise BAG2 in complex with the 15 kDa protein later identified as cytochrome *b*_562_.

Initially, BAG2 expression was performed according to Mukherjee *et al.* (2015 ▸, 2020 ▸) utilizing the *E. coli* BL21-Gold strain. Subsequent Protein-L and cation-exchange steps yielded a homogenous sample comprising the ∼25 kDa heavy (HC) and light chains (LC) of BAG2 and an unexpected species of ∼15 kDa, as indicated by SDS–PAGE (Figs. 1 ▸*a* and 1 ▸*c*). Expression of BAG2 in alternative *E. coli* expression strains [Rosetta (DE3), C43 (DE3) Pro^+^ and T7 Express] yielded identical results.

Originally, we presumed this ∼15 kDa species to arise from proteolytic degradation of BAG2; however, this seemed unlikely given that both species eluted from cation exchange within the same sharp peak (Fig. 1 ▸*b*). Therefore, we proposed the ∼15 kDa band to indicate a co-purifying protein with high affinity for BAG2. This notion was supported via size-exclusion chromatography, which yielded two distinct species following incubation of our purified BAG2 sample with a BRIL fusion protein of ∼37 kDa (Fig. 1 ▸*d*). SDS–PAGE indicated the larger species to comprise the expected BRIL fusion protein–BAG2 complex; however, the ∼15 kDa protein retained in a complex with a substantial proportion of BAG2, eluting as a second, smaller complex (Figs. 1 ▸*d* and 1 ▸*e*).

Both our purified BAG2 sample and SEC fractions comprising the ∼15 kDa protein exhibited a distinct red colour suggestive of an iron-containing molecule, whilst those corresponding to the desired BRIL fusion protein–BAG2 complex were clear. We therefore concluded that BAG2 was likely co-purifying with the native cytochrome *b*_562_ (hereafter referred to as cyt *b*_562_) present within the *E. coli* periplasm (Itagaki & Hager, 1966 ▸). Indeed, BRIL is itself a thermostabilized derivative of apocytochrome *b*_562_ (Chun *et al.*, 2012 ▸); thus, the ability of the native cytochrome to compete with BRIL for BAG2 binding is unsurprising.

### 3.2. Determination of the cytochrome *b*_562_–BAG2 complex

**Figure 2:**
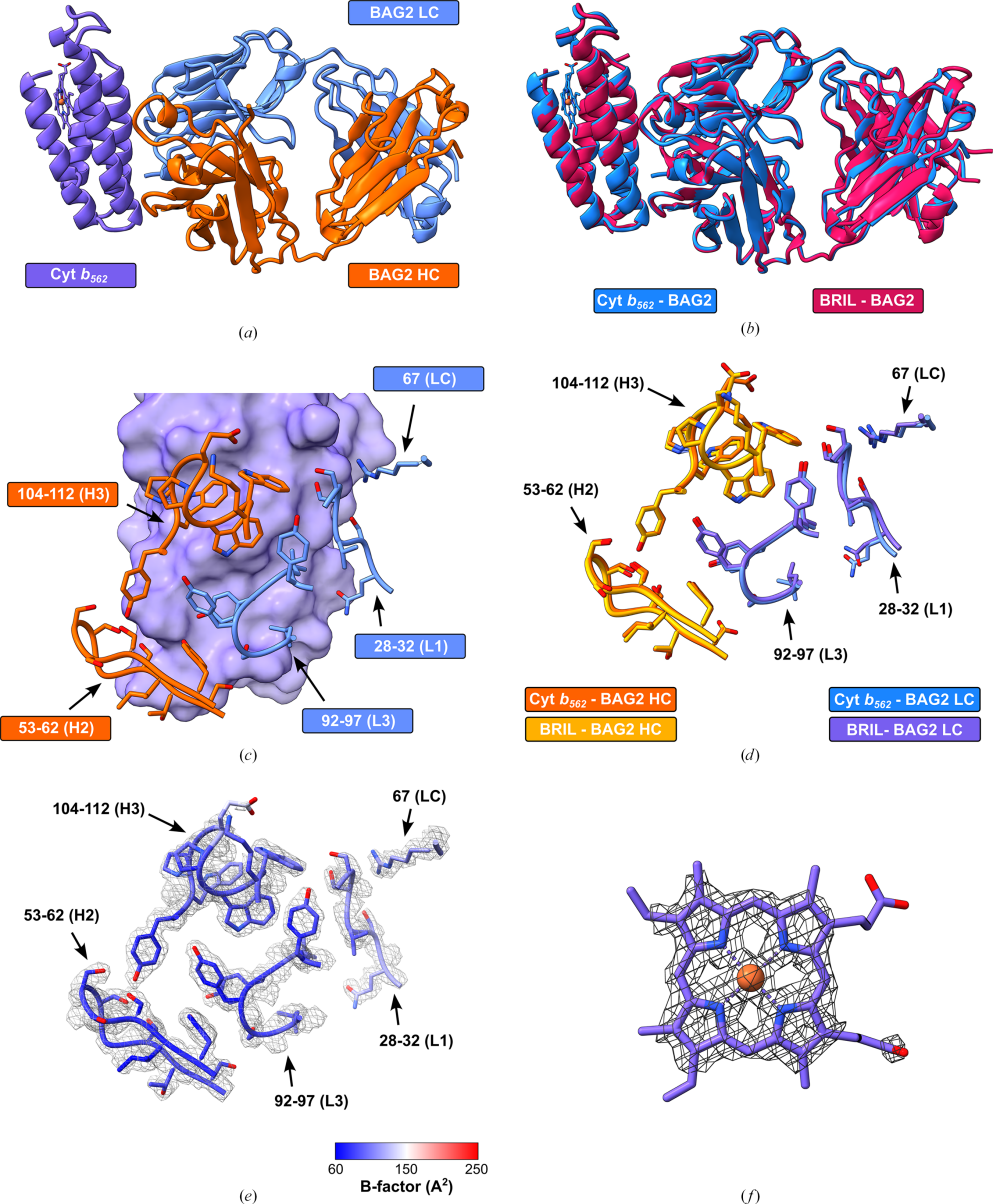
Crystal structure of BAG2 in complex with cytochrome *b*_562_. (*a*) Crystal structure of the cytochrome *b*_562_–BAG2 complex in cartoon representation. (*b*) Alignment of our cyt *b*_562_–BAG2 structure (blue) with the previously determined BRIL–BAG2 structure (pink; PDB entry 6cbv). (*c*) Interaction of BAG2 (heavy and light chains in orange and blue, respectively) with cytochrome *b*_562_ (depicted as a purple surface). Brackets denote the contributing region of BAG2. (*d*) Overlaid CDR binding modes of BAG2 in complex with cytochrome *b*_562_ and BRIL. Heavy and light chains are coloured orange and blue, respectively, for the cytochrome *b*_562_-bound structure and gold and purple, respectively, for the BRIL-bound structure (PDB entry 6cbv). (*e*) Refined electron density for the BAG2 CDR regions interacting with cytochrome *b*_562_. The 2*mF*_o_ − *DF*_c_ map (grey mesh) is contoured at 1σ and BAG2 is displayed in ball-and-stick representation, coloured by *B* factor. (*f*) Refined electron density for the cytochrome *b*_562_ heme group. The 2*mF*_o_ − *DF*_c_ map (grey mesh) is contoured at 1σ and the heme group is displayed in ball-and-stick representation.

To confirm our hypothesis that BAG2 was co-purifying with the native cyt *b*_562_, we sought to solve the structure of their complex. The cyt *b*_562_–BAG2 complex crystallized in space group *P*6_5_ and diffracted to 2.65 Å resolution, with one copy of each protein in the asymmetric unit (Fig. 2 ▸*a*), resulting in a Matthews coefficient of 3.15 Å^3^ Da^−1^ (Matthews, 1968 ▸) and a corresponding solvent content of 61%.

As expected, our cyt *b*_562_–BAG2 complex exhibited an almost identical architecture to that of the previously educated BRIL–BAG2 complex (PDB entry 6cbv), with alignment yielding an r.m.s.d. of 0.539 Å across 529 pruned atom pairs and 0.686 Å across all 539 pairs (Fig. 2 ▸*b*). Furthermore, the BAG2 complementarity-determining regions (CDRs) exhibit the same binding mode in both structures, in which both CDR-H2 and CDR-H3 supplement extensive interactions made by the LC CDRs (Figs. 2 ▸*c* and 2 ▸*d*). The average *B* factors for our cyt *b*_562_–BAG2 complex are relatively high (109 Å^2^), likely arising due to the high solvent content of the crystal; nevertheless, the electron density for the BAG2 CDRs was well defined, allowing accurate modelling of their interaction with cyt *b*_562_ (Fig. 2 ▸*e*). Furthermore, unambiguous density was observed for the heme molecule within the cytochrome (Fig. 2 ▸*f*). Our cyt *b*_562_–BAG2 crystal structure demonstrates the ability of BAG2 to form a stable complex with the native cytochrome *b*_562_ present within canonical *E. coli* expression strains.

### 3.3. Generation of a Δ*cybC* strain for bacterial BAG2 expression

Given the propensity of BAG2 to bind native cytochrome *b*_562_, we investigated possible methods to prevent their co-purification entirely. We noted the superfluous nature of cyt *b*_562_ given that disruption of the *cybC* gene prevents its expression in *E. coli* K strains (Trower, 1993 ▸). Thus, we hypothesized that deletion of the *cybC* gene from a typical B-lineage *E. coli* expression strain would directly enable contaminant-free BAG2 expression and purification.

**Figure 3:**
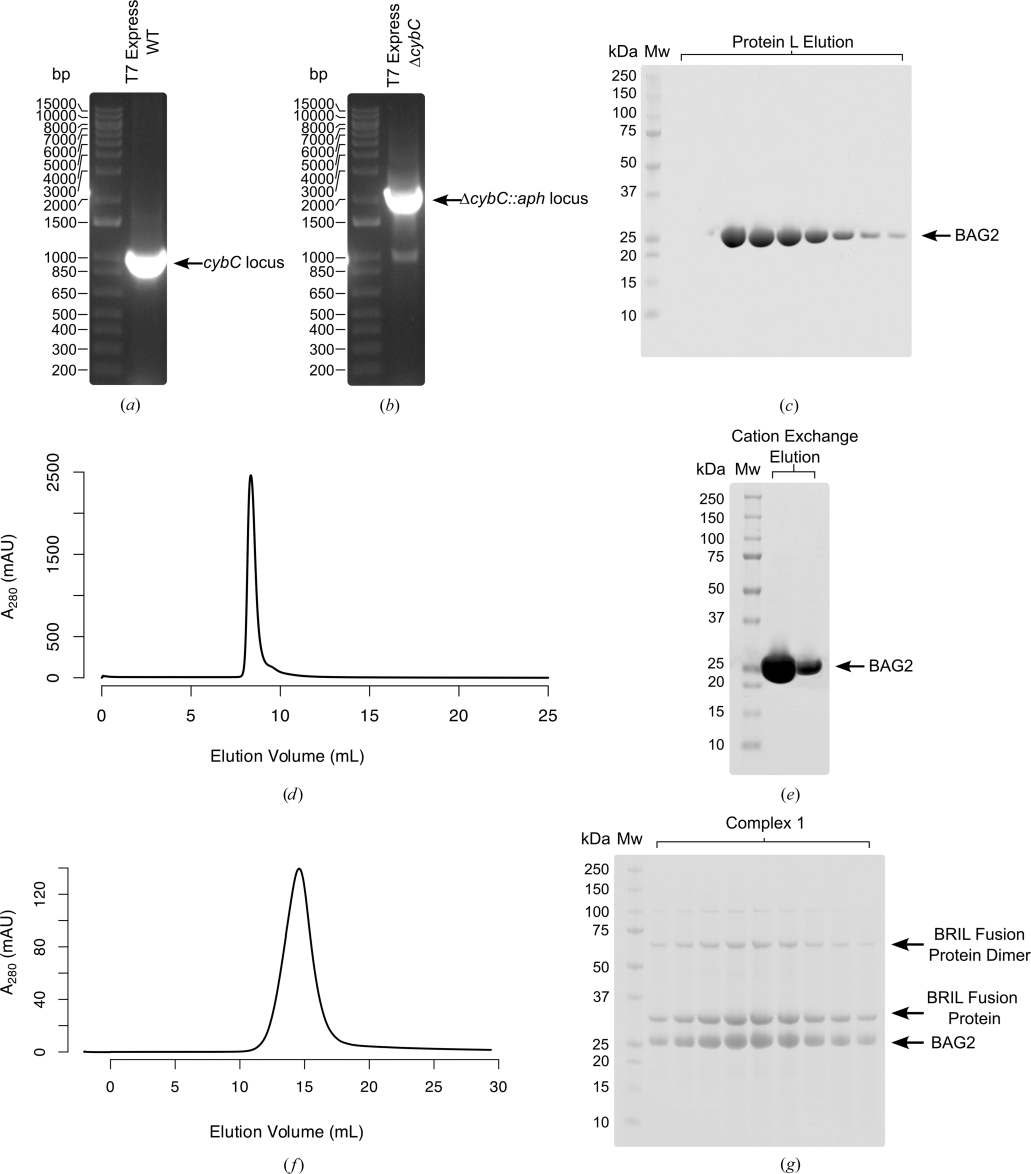
Generation of a Δ*cybC* strain for bacterial BAG2 expression. (*a*) Gel electrophoresis of colony PCR amplifying the WT T7 Express *cybC* locus using flanking primers. (*b*) Gel electrophoresis of colony PCR amplifying the T7 Express Δ*cybC::aph* locus using flanking primers. (*c*) SDS–PAGE of Protein L elution fractions following BAG2 overexpression in T7 Express *ΔcybC* indicating no co-purifying proteins. (*d*) Cation-exchange trace of pooled Protein L elution fractions following BAG2 overexpression in T7 Express *ΔcybC.* (*e*) SDS–PAGE of cation-exchange fractions following BAG2 overexpression in T7 Express *ΔcybC*. (*f*) Size-exclusion chromatography trace following incubation of a BRIL fusion protein (∼37 kDa) with BAG2 purified from T7 Express *ΔcybC* indicating complete formation of the desired complex. (*g*) SDS–PAGE of the size-exclusion chromatography fractions following incubation of a BRIL fusion protein (∼37 kDa) with BAG2 purified from T7 Express *ΔcybC*.

Utilizing Datsenko and Wanner gene inactivation, we replaced the *cybC* ORF within the *E. coli* T7 Express strain with a kanamycin cassette and subsequently P1-transduced the mutant allele into a fresh background (Figs. 3 ▸*a* and 3 ▸*b*). Expression utilizing this engineered strain, followed by the same Protein L and cation-exchange purification, yielded a pure BAG2 sample (Figs. 3 ▸*c*, 3 ▸*d* and 3 ▸*e*) that formed only the desired BRIL fusion protein–BAG2 complex (Figs. 3 ▸*f* and 3 ▸*g*). Indeed, in our hands, BAG2 was sufficiently clean following Protein L purification that the elution fractions may be dialysed directly, avoiding the necessity for a subsequent cation-exchange step (Fig. 3 ▸*c*).

## 4. Discussion and conclusions

The use of monoclonal fragments antigen binding (Fabs) is a prevalent methodology facilitating protein structure determination via both crystallography and cryo-EM. The development of a synthetic Fab against the BRIL domain improved accessibility to this approach, providing a general fiducial applicable to any protein of interest via the simple curation of a BRIL fusion protein (Mukherjee *et al.*, 2020 ▸).

We observed BAG2 to co-purify with the native *E. coli* cytochrome *b*_562_, which hindered the assembly of our desired BRIL fusion protein–BAG2 complex (Figs. 1 ▸*d* and 1 ▸*e*). Consequently, we constructed a T7 Express Δ*cybC* strain, allowing the production of a pure BAG2 sample from a simple Protein L purification (Fig. 3 ▸*c*).

Nonetheless, we emphasize that several publications have previously expressed and purified BAG2 from unmodified *E. coli* strains without issue (Mukherjee *et al.*, 2020 ▸; Sverak *et al.*, 2024 ▸; Tsutsumi *et al.*, 2020 ▸); thus, the maintained inter­action between BAG2 and cyt *b*_562_ appears to result from minor differences during purification. Indeed, Mukherjee *et al.* (2020 ▸) recommend incubation of the bacterial lysate at 60–63°C for 30 min to denature bacterial proteins and Fab degradation products. In contrast, our incubation at 58°C for the same duration appears to be insufficient to completely dissociate the unwanted complex, implying the incubation temperature to be a critical factor in ensuring efficacious purification of BAG2 from canonical *E. coli* expression strains.

Nevertheless, our T7 Express Δ*cybC* strain offers an alternative bacterial system for the expression of BAG2, which completely prevents its association with cytochrome *b*_562_ whilst also providing an efficient and affordable substitute to the insect and mammalian systems also utilized (Dang *et al.*, 2022 ▸; Kugawa *et al.*, 2025 ▸). Whilst we demonstrated the efficacy of our Δ*cybC* strain for BAG2, we speculate that it may also provide a useful resource for the expression of any previously developed synthetic anti-BRIL Fab (BAG5, BAK5 and BAK7; Mukherjee *et al.*, 2020 ▸).

## Supplementary Material

PDB reference: native cytochrome *b*_562_ in complex with the synthetic anti-BRIL antibody BAG2, 9tmp

## Figures and Tables

**Table 1:** Crystallization of the native cytochrome *b*_562_–BAG2 complex

Method	Sitting-drop vapour diffusion
Plate type	MRC 2-lens
Temperature (K)	277
Protein concentration (mg ml^−1^)	10
Buffer composition of protein solution	20 m*M* Tris, 150 m*M* NaCl, 0.02% DDM pH 8
Composition of reservoir solution	0.1 *M* MOPS/HEPES–Na pH 7.5, 0.03 *M* bromide, 0.03 *M* fluoride, 0.03 *M* imidazole, 12.5%(*w*/*v*) PEG 1000, 12.5%(*w*/*v*) PEG 3350, 12.5%(*v*/*v*) MPD
Volume and ratio of drop	100 nl protein plus 100 nl reservoir (1:1)
Volume of reservoir (µl)	40
Composition of cryoprotectant	Reservoir solution

**Table 2:** Data collection and processing of the native cytochrome *b*_562_–BAG2 complex Values in parentheses are for the highest resolution shell.

PDB code	9tmp
Beamline	I04, DLS
Wavelength (Å)	0.9537
Temperature (K)	100
Detector	EIGER2 XE 16M
Total rotation range (°)	360
Rotation per image (°)	0.1
Exposure time per image (s)	0.0071
Space group	*P*6_5_
*a*, *b*, *c* (Å)	88.72, 88.72, 162.51
α, β, γ (°)	90, 90, 120
Mosaicity (°)	0.087
Resolution range (Å)	76.84–2.64 (2.68–2.64)
Total No. of reflections	441423 (14769)
No. of unique reflections	21369 (1061)
Completeness (%)	100 (100)
Multiplicity	20.66 (13.92)
〈*I*/σ(*I*)〉	11.18 (0.17)
CC_1/2_	0.999 (0.119)
*R*_p.i.m._	0.036 (1.922)
Overall *B* factor from Wilson plot (Å^2^)	74.47

**Table 3:** Structure refinement of the native cytochrome *b*_562_–BAG2 complex Values in parentheses are for the highest resolution shell.

Resolution range (Å)	44.28–2.65 (2.79–2.65)
Completeness (%)	91.64 (42.28)
No. of reflections, working set	19287 (1277)
No. of reflections, test set	1008 (56)
Final *R*_cryst_	0.2077 (0.4017)
Final *R*_free_	0.2414 (0.4344)
No. of non-H atoms	
Macromolecules	4106
Ligands	43
Solvent	1
Total	4150
R.m.s. deviations	
Bond lengths (Å)	0.008
Angles (°)	0.9
Average *B* factors (Å^2^)	
Macromolecules	109.78
Ligands	128.52
Solvent	66.98
Ramachandran plot	
Favoured regions (%)	97.57
Allowed (%)	2.43
Outliers (%)	0

**Table 4:** Primers utilized in this study

Primer name	Primer sequence (5′–3′)
oBFC307	GTGAAGGATGAAGTGTAAATAAAAAAGGAAGTGAGCAATGCATATGAATATCCTCCTTAG
oBFC308	GCAACAGGGAAATGAGGAATTAACGATACTTCTGGTGATAGTGTAGGCTGGAGCTGCTTC
oBFC309	CCCGTTAGTGAAATCACCATCGCAG
oBFC310	AGGGTATTTCCCTCTCCGGCG

## Data Availability

The structure factors and model of the native cytochrome *b*_562_–BAG2 complex have been deposited in the Protein Data Bank (PDB entry 9tmp).
